# Rationale, design and methods for a randomised and controlled trial of the impact of virtual reality games on motor competence, physical activity, and mental health in children with developmental coordination disorder

**DOI:** 10.1186/1471-2458-11-654

**Published:** 2011-08-18

**Authors:** Leon M Straker, Amity C Campbell, Lyn M Jensen, Deborah R Metcalf, Anne J Smith, Rebecca A Abbott, Clare M Pollock, Jan P Piek

**Affiliations:** 1 School of Physiotherapy, Curtin University, Perth, Australia; 2 Curtin Health Innovation Research Institute, Curtin University, Perth, Australia; 3 School of Psychology, Curtin University, Perth, Australia; 4 School of Human Movement Studies, The University of Queensland, Brisbane, Australia

## Abstract

**Background:**

A healthy start to life requires adequate motor development and physical activity participation. Currently 5-15% of children have impaired motor development without any obvious disorder. These children are at greater risk of obesity, musculoskeletal disorders, low social confidence and poor mental health. Traditional electronic game use may impact on motor development and physical activity creating a vicious cycle. However new virtual reality (VR) game interfaces may provide motor experiences that enhance motor development and lead to an increase in motor coordination and better physical activity and mental health outcomes. VR games are beginning to be used for rehabilitation, however there is no reported trial of the impact of these games on motor coordination in children with developmental coordination disorder.

**Methods:**

This cross-over randomised and controlled trial will examine whether motor coordination is enhanced by access to active electronic games and whether daily activity, attitudes to physical activity and mental health are also enhanced. Thirty children aged 10-12 years with poor motor coordination (≤ 15^th ^percentile) will be recruited and randomised to a balanced ordering of 'no active electronic games' and 'active electronic games'. Each child will participate in both conditions for 16 weeks, and be assessed prior to participation and at the end of each condition. The primary outcome is motor coordination, assessed by kinematic and kinetic motion analysis laboratory measures. Physical activity and sedentary behaviour will be assessed by accelerometry, coordination in daily life by parent report questionnaire and attitudes to physical activity, self-confidence, anxiety and depressed mood will be assessed by self report questionnaire. A sample of 30 will provide a power of > 0.9 for detecting a 5 point difference in motor coordination on the MABC-2 TIS scale (mean 17, sd = 5).

**Discussion:**

This is the first trial to examine the impact of new virtual reality games on motor coordination in children with developmental coordination disorder. The findings will provide critical information to understand whether these electronic games can be used to have a positive impact on the physical and mental health of these children. Given the importance of adequate motor coordination, physical activity and mental health in childhood, this project can inform interventions which could have a profound impact on the long term health of this group of children.

**Trial registration:**

Australia and New Zealand Clinical Trials Register (ANZCTR): ACTRN12611000400965

## Background

### Computer use by children is a major change in our society

Nearly all Australian children now use computers and video games [1]. More than 79% of households with children have a computer and more than 60% have a video game machine [2]. A recent meta analysis of studies in affluent countries found boys' and girls' mean computer/video game use was 74 minutes a day [3]. Electronic game use is increasing rapidly, with Roberts et al. [4] reporting a doubling since the meta analysis studies.

In a recent review we [5] reported that the available evidence suggested computer use targeted on learning areas is associated with enhanced academic achievement (e.g. [6]) but that electronic game playing has a negative effect on school achievement [7]. We also found that game-related discourse may provide a stimulus for children's social development [8], although there are concerns about the potential negative effects of violence in electronic games [9].

Research on the impact of computer use on children's physical development has focused on postures during computer use at school [10], use of laptop computers [11] and the impact of workstation design on posture and muscle activity [12]. Whilst this research has suggested potential musculoskeletal problems associated with prolonged and constrained postures and repetitive small movements, there is no evidence available on the impact of computer or electronic game use on motor development. We have raised concerns that electronic game use may have a negative impact on gross motor development as it may displace other childhood leisure activities which provide critical practice of gross motor tasks and which facilitate motor development [5].

### Electronic game use may have a negative impact on normal motor development

Normal motor development requires maturation of neural and muscular systems plus the opportunity to practise fine and gross motor skills, with studies on children with impoverished or enriched motor environments providing evidence for the importance of practice [13]. Gross motor experiences are usually associated with physical activity (PA) (defined as the movement of the limbs and torso by muscle activity resulting in energy expenditure). There is evidence that increased PA can provide the practice necessary to improve gross motor skill development in children with normal motor development [14]. However there are concerns amongst researchers and parents that electronic game playing reduces children's PA.

Traditional electronic game interfaces can provide motor experience, but fine rather than gross. Yuji [15] reported evidence that electronic games improved children's fine motor performance. In a review, Whitcomb [16] found electronic game playing lead to enhanced eye-hand coordination, dexterity and fine motor ability and increased reaction and movement speeds in elderly subjects and Rosser et al. [17] found a dose response relationship between video game experience and laproscopic surgery training performance (both speed and accuracy).

In contrast, traditional electronic game playing probably does not provide gross motor experience, and may lead to a decline in gross motor skill. In an epidemiological study of 1,600 five-year-olds we found that computer use did displace vigorous PA on weekends reducing overall gross motor experience [18] and suggesting a potential vicious cycle.

### Children with DCD are at greater risk

**Figure 1:**
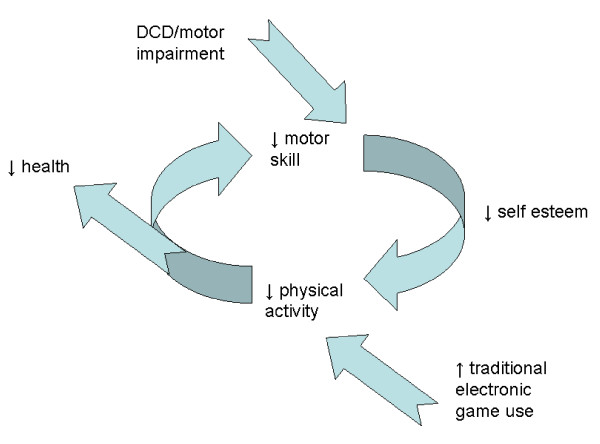
**Vicious cycle of physical activity, motor skill and self esteem**.

Five to 15% of children have developmental coordination disorder (DCD), defined as lacking developmental, age-appropriate motor skills, and characterised by motor performance impairment that creates functional performance deficits not likely to be due the child's age, intellect, or other diagnosable neurological or psychiatric disorder [19,20]. Underlying deficits identified in children with DCD include poor sensory-motor integration [21] and in particular, poor visuomotor processing [22], or cross-modal integration (visual-kinaesthetic). Children with DCD also have poor timing and force control and it has been argued that there may be a disruption in the central timing mechanisms, usually linked with cerebellar function [23]. A recent review found that these children are less physically active and have lower levels of fitness [24]. Children with DCD are thus at greater risk of insufficient PA [25] and a downward spiral of poorer motor development, psychological and health outcomes (Figure 1).

### Motor skill can be improved in children with DCD

Whilst there are varying views as to the underlying pathology of DCD and no consensus on the correct approach to intervention, a recent review concluded that there is sufficient evidence of good quality to confirm that interventions are effective in improving motor skill in children with DCD [26]. Approaches ranging from perceptual-motor therapy, to sensory-integration therapy, mastery and physiotherapy are supported with little evidence of the superiority of any specific approach [26], although studies were of variable quality. Intervention doses range from 30 minutes weekly for 6 weeks [27] to 50 minutes 3 times a week for 10 weeks [28].

### Motor skill is important for physical activity participation

Children need to have a certain degree of motor skill to feel confident to engage in PA. Harter's [29,30] competence motivation theory argues that children's motivation to engage in an activity (in this case, PA) is determined partly by the confidence they have in their ability to carry out these tasks. Hence, children who are more skilled would be more likely to engage in the activity. According to Doganis and Theordarakis [31], attitude theory best explains engagement in PA and sport. It is considered to comprise three elements, namely cognitive, affective and behavioural. A child's perception of their own ability is one component of the cognitive element. Their enjoyment of the activity, or the anxiety it generates, are part of the affective element. The behaviour component relates to the outcomes, good or bad, of participation and its reinforcement qualities. Therefore a vicious circle may develop for children where reduced PA results in reduced motor skill which further impedes PA participation.

### Physical activity is important for health and development

Over 80% of Australian children do not meet national standards for adequate PA [32]. A lack of adequate PA has been linked with childhood health issues including obesity, bone mineral density, type II diabetes and cardiovascular risk factors [25,33]. Boys with DCD appear to be at greater risk of obesity [34].

Children with DCD have been found to engage less in PA than other children [25]. More recently Poulsen et al. [35] found a negative correlation between DCD and participation in social PA. It has been suggested that this is due to the fact that children with DCD do not like to display their poorer athletic skill to their peers [34], in line with Harter's [29,30] competence motivation theory. Our research [36] has demonstrated that children with DCD perceive themselves as poorer in the domain of athletic competence, and this has been linked to high levels of anxiety [37] and depressive symptomatology [38]. In line with attitude theory, this negative affect would then impact on the child's future interest in engaging in PA.

### Electronic games have traditionally been sedentary

Electronic games have traditionally used keyboard/mouse and game pad interfaces which require very little movement. In a laboratory study of children with normal motor development we found energy expenditure during traditional electronic game playing to be similar to watching a DVD [39]. We also found minimal levels of muscle activity and movement during traditional electronic game playing [40]. Whilst traditional electronic games may have some positive physical impact on fine motor skills this may be offset by decreasing overall PA levels and reduced gross motor practice. Poorer PA outcomes may be associated with lower activity during actual game playing and by the displacement of more active leisure activities. Reduced gross motor practice may lead to poorer motor development. Whilst Li and Atkins [41] found some evidence of a relationship between poorer gross motor ability and increasing computer use in pre schoolers there is no direct evidence on the link between electronic games and poorer motor skills.

Marshall et al. [42] reviewed available studies and found 10 cross sectional studies showing a weak negative relationship (r = -0.14) between electronic games/computer use and PA. However the nature of computer use was not specified so included games and other uses. Recent reviews have included longitudinal studies and have also questioned whether electronic game use displaces more vigorous PA [43,44]. Despite no experimental evidence of a causal effect of electronic games on motor development, attitudes to PA or overall PA there is considerable community concern.

**Figure 2:**
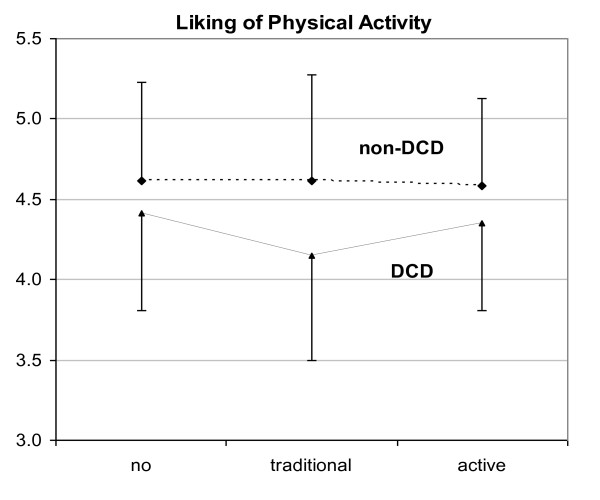
**Liking of physical activity ratings in children with and without developmental coordination disorder following 8 weeks with no electronic games, 8 weeks with traditional electronic games and 8 weeks with active VR games**.

In a recent pilot study with 12 children (6 with DCD) [45] we found access to traditional electronic games for 8 weeks resulted in a trend for a 23.1% reduction in accelerometer assessed energy expenditure on non school days (p = .122) and significant reductions in both motor competence (McCarron Assessment of Neuromotor Disorders Neurodevelopmental Index [46] 7.3% p = .044) and liking of physical activity [47] (6.0% p = .048) compared to an 8 week period when electronic games were removed from the house of children with DCD (see Figure 2).

### New opportunities with active virtual reality games

Virtual reality (VR) refers to a simulated interactive environment. VR aims to create a visual, auditory and sometimes tactile and olfactory environment that appears real and enables the human user to become immersed in the interactive experience. VR has been extensively used in commercial/military training applications such as flight and surgery simulators. It has also been used for arcade games and recently for patient rehabilitation. Until recently the only VR systems were expensive ($100,000+) laboratory based systems or large dedicated leisure simulators. However cheap (< $500) VR systems such as Eye and Move (Sony), Wii (Nintendo) and Kinect (Microsoft) have been released, making VR available to households.

Video capture VR (Eye, Kinect) uses a video camera to capture the user's image and movement and embed this into the virtual environment. Whilst it lacks haptic response and single camera systems (Eye) can only track movement in one plane, it requires no head mounted display or exoskeletion thus enabling movement free of encumbrance. Dual camera systems (Kinect) can track in three dimensions. It also provides a mirror view enabling immediate feedback on posture and quality of movement. Users report the interaction is intuitive and natural, with ratings of sense of presence and enjoyment equalling those of expensive laboratory based systems [48].

### VR electronic games may lead to improved motor skill and PA outcomes

A critical feature of video capture VR games is that it requires arm, leg or whole body movement. It may therefore provide gross motor experiences that are not available when interacting using traditional interfaces such as keyboard, mouse or game pad. Video capture VR may thus enable children to play electronic games without the previously observed detrimental physical effects.

We and others have recently reported significant increases in energy expenditure, heart rate and ventilation volume when children played a game with video capture VR compared to a traditional interface [39,49,50]. Further, trials are being conducted to determine whether VR games in the home can enhance health outcomes for children [51,52].

VR electronic games can enhance motor skill in adults following brain injury with improved locomotion, upper and lower extremity function [53]. VR has demonstrated some improvements in motor performance in case studies of children with cerebral palsy [54]. Wann et al. [55] argue that VR is an ideal tool for remedial programs involving attention and movement disorders, and discuss its use in the context of stroke patients. VR may be particularly successful for children with DCD as it does not require the child to perform in front of other children. Lack of PA in children with DCD has been attributed to their unwillingness to display their poor skill to others. However, VR electronic games may improve these children's skill by providing gross motor practice involving a high level of visual-spatial integration, but in a context which is private, and provides strong motivation by enjoyment of the game and the challenge of self-competition. However this will only occur if the nature of the movement required is suitable.

Improvements in performance in VR are useful if they lead to improvements in real world performance. Whilst there is no available data on this in children, there is evidence of balance gains from VR training resulting in improved real world balance in elderly subjects undergoing rehabilitation [53]. VR training also leads to greater enjoyment of rehabilitation and improved motor confidence in the real world in adults. This suggests VR games could improve real world motor skill in children and could increase children's confidence, which would be additionally beneficial for children with DCD. However, there is no evidence of the effect of VR on children with DCD.

**Figure 3:**
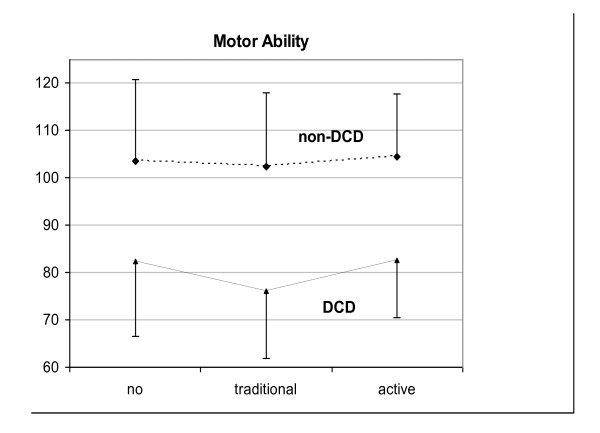
**Motor ability (MAND NDI) in children with and without developmental coordination disorder following 8 weeks with no electronic games, 8 weeks with traditional electronic games and 8 weeks with active VR games**.

In our recent pilot study involving 6 children with DCD we found access to video capture VR games for 8 weeks tended to increase motor competence (MAND NDI 8.8%, p = .041) as well as accelerometer measured energy expenditure on non school days (54.3% p = .093), compared with access to traditional electronic games in children with DCD and no apparent effect on children without DCD (see Figure 3).

### Study Aim

Therefore the main aim of this study is to determine whether access to new high fidelity active VR electronic games can enhance motor coordination in children with motor impairment. Secondary aims include investigating whether increases in VR performance translate to increases in real world gross motor performance and increases in physical activity, attitudes to physical activity, self confidence and mental health.

## Methods/design

### Design and Aims

This study will use a cross over randomised controlled trial to assess the impact of active electronic game use on motor coordination and physical and mental health correlates in children with DCD by:

1) Comparing change in motor coordination over 16 weeks when children use active electronic games or no active electronic games. We hypothesise that motor coordination will improve more when children use active electronic games.

2) Comparing change in parent reports of motor coordination during activities of daily living over 16 weeks when children use active electronic games or no active electronic games. We hypothesise that parent reports of motor coordination will improve more when children use active electronic games.

3) Comparing change in physical activity and sedentary behaviour over 16 weeks when children use active electronic games or no active electronic games. We hypothesise that moderate/vigorous physical activity will increase and sedentary time will decrease more when children use active electronic games.

4) Comparing change in attitudes to physical activity over 16 weeks when children use active electronic games or no active electronic games. We hypothesise that attitudes to physical activity will improve more when children use active electronic games.

5) Comparing change in child reports of mental health over 16 weeks when children use active electronic games or no active electronic games. We hypothesise that mental health will improve more when children use active electronic games.

### Sample

30 children (10-12 years of age) will be recruited by mass media, university and school notices and professional networks. This age group has been selected as they are able to provide detailed information in diary and questionnaires, have a high use of electronic games and are developing physical and mental behaviour patterns pre-puberty which may track into adulthood. Children of parents expressing interest will be screened to ensure they are willing to participate after being informed of the full study responsibilities and meet the inclusion and exclusion criteria. Inclusion criteria are: aged 10-12 years at start of study, able to use electronic games on most days and being classified as DCD. DCD status will be assessed using Movement Assessment Battery for Children-2 (MABC-2 [56]. The MABC-2 comprises 8 tasks, three measuring manual dexterity, 3 measuring aiming and catching and 2 measuring balance. Age norms based on a standardisation sample of 1,172 children are used to determine a standard total score (*M *= 10, *SD *= 3). Separate standard scores can be determined for each of the sub-tests. In addition to the total score, a set of qualitative observations allows the examiner to record the child's performance characteristics during the testing. Cut-offs for impairment scores are at or below the 5th percentile for definite motor difficulties, whilst scores above the 5th percentile but at or below the 15th percentile suggest borderline difficulties. The 15^th ^percentile cut off will be used in this study as this is recommended for research purposes to prevent excluding children with mild DCD [57]. Minimum value of the test-retest reliability of the original MABC is 0.75 and the inter-tester reliability is 0.70. The original MABC has been found to correlate well with other movement tests [58,59]. The MABC-2 will be conducted at a location agreed to by parents, typically the child's home. Children will be excluded if they have a diagnosed disorder likely to impact their study participation, movement or electronic game use (other than developmental coordination disorder), live in a shared care arrangement where the child spends a significant amount of time in different houses and is unable to maintain game access condition, or live remote to the University campus. The child's age and sex will be recorded at baseline along with their experience with electronic games and computers using the Young people's Activity Questionnaire [11].

For power calculations, motor impairment (MABC-2 Total Impairment Score) was estimated at 17+5 with a minimum effect size of 5 considered important based on effects in prior studies [60]. If the variation in the motor impairment between repeated time points in each individual is normally distributed with standard deviation 5, and the true effect of game condition is 5, a study with 30 subjects will reject the null hypothesis that this response difference is zero with probability (power) 0.9027. The Type I error probability associated with this test of this null hypothesis is 0.01.

Volunteers and their parents will be provided with a detailed written description of the study purpose, procedures, risks and benefits and given an opportunity to ask research staff for clarification prior to signing assent (children) and consent (parents) to participate. The study has ethical approval from the Human Research Ethics Committee of Curtin University (approval number HR11/2011).

### Intervention and control conditions

There will be two levels of electronic game access. 'No active electronic games' will involve all active input electronic games being removed from the family home with a contract that active electronic games will be avoided where possible at other locations. Participants will be able to play traditional electronic games using a game pad input during this period. 'Active electronic games' will involve the provision of a Sony PlayStation 3 with Move and Eye input devices and Microsoft Xbox360 with Kinect input device and a range of non-violent games. Children will be contacted regularly during the study and asked to report exposure to electronic games including games played, frequency and duration and level achieved. Children will also be asked about participation in other activities such as sports and hobbies.

A condition period of 16 weeks will allow time for improvements in motor impairment during the active electronic games condition whilst fitting in with school holiday schedules. A within subjects design allows reduced study numbers.

### Outcome measures

#### Motor coordination

Motor coordination will be assessed using the MABC-2 and during a series of tasks performed in a motion analysis laboratory. Detailed kinematic and kinetic data will be collected using a three-dimensional motion analysis system (Vicon; Oxford Metrics, inc.) and two AMTI force plates (Advanced Mechanical Technology, inc.). The large (0.6 * 1.2 m) and small (0.6*0.3 m) force plates are located in the middle of the laboratory directly next to one another and will be operated at 1000 Hz. The 14 camera Vicon motion analysis system will be calibrated to collect data in an approximately 5 m^2 ^area in the middle of the laboratory, running at 250 Hz. Prior to trial performance each child will be fit with the custom full body marker set (seventy two 14 mm retro-reflective markers). This marker set (Table 1) follows a cluster based protocol and allows the accurate calculation of full body joint kinematics and kinetics complicit with the International Society of Biomechanics recommendations [61,62].

**Table 1:** Three-dimensional motion analysis marker set

Marker	Anatomical location	Real (R) or Virtual (V)*
**Head markers**		

Eye markers	Placed lateral to canthus of the left and right eyes	R

Ear markers	Placed above the tragus (or concha) of the left and right ears	R

Nose marker	Placed on the tip of the nose	V

**Thorax/Back markers**		

Anterior thorax marker	Sternal notch between the two clavicles	R

Posterior thorax markers	Placed on the 7^th ^cervical and 6^th ^thoracic vertebrae	R

**Pelvis:**		

Anterior pelvis	Right and left anterior superior iliac crests	R

Posterior pelvis	Right and left posterior superior iliac crest	R

**Lower limbs**		

Thigh markers	Three markers set on a t-bar cluster, with the long bar fixed mid-segment along the iliotibial band. The short bar raps medially onto the quadriceps.	R

Tibia markers	Three markers set on a t-bar cluster, with the long bar fixed mid-segment along the tibia. The short bar raps laterally towards the fibula.	R

Knee markers	Four markers placed on the right and left, medial and lateral femoral condyles	V

Feet markers	Three markers placed on the calcaneus, talus hook and 5^th ^metatarsal	R

Ankle markers	Four markers placed on the right and left, medial and lateral malleoli	V

**Upper limbs**		

Shoulder markers	Right and left, anterior and posterior shoulder markers	V

Right and left acromion markers	Three markers placed on the posterior and anterior portion of the lateral border of the acromial plateau, with one marker placed laterally at the base of the acromioclavicular joint.	R

Right and left upper arm markers	Three markers set on a t-bar cluster, with the long bar fixed mid-segment on the lateral aspect of the upper arm. The short bar raps laterally towards the biceps.	R

Elbow markers	Right and left, medial lateral epicondyle markers	V

Forearm markers	Three markers placed on the medial and lateral aspect of the distal third of the forearm, with one marker placed mid segment - between the radius and ulnar, midway up the forearm.	R

Wrist markers	Right and left, medial and lateral wrist markers placed on the ulnar and radial styloid processes	V

Hand markers	Three markers, two placed mid-hand medially and laterally. The third marker is placed directly below the junction between the third metacarpal and third proximal phalange.	R

Finger markers	Two smaller markers (5 mm diameter) fixed to the most distal portion of the index finger, finger nail	R

**Equipment**		

Ball markers	Three markers fixed to each ball; tennis ball, t-ball ball, soccer ball	R

T-ball bat tip	A single marker fixed to the tip of the t-ball bat	R

* 'Virtual' markers are removed after one static trial. 'Real' markers remain throughout the motion analysis data collection

Three tasks drawn from common motor performance tools will be used to assess whole body coordination: running, single leg stance and horizontal jump. Running will be performed using the Test of Gross Motor Development, 2^nd ^edition (TGMD-2; [63]) protocol which involves running over the 10 meter laboratory runway with children instructed to run as fast as they can. Five trials will be performed. Trials will be deemed successful if the child strikes one of the force platforms with their preferred leg. The starting point of the runway will be adjusted in the instance successful foot strike does not occur and the child will not be informed of this requirement to facilitate natural running technique. Single leg stance will be performed using the MAND [46] protocol which involves balancing on their preferred foot with the arms free to move for balance, the unused leg held off the floor with slight knee flexion and pivoting allowed but hopping not. Children will be instructed to stand on the large force plate on their preferred foot for as long as they can or until told to stop. Two trials will be completed with a maximum period of 30 seconds for each trial. Horizontal jump will be performed using the TGMD-2 protocol which involves jumping with feet parallel at start and finish. Children will be instructed to jump as far as they can, while 'sticking' their landing and will perform 5 trials. The take-off will be performed from the middle of the small force plate.

Four tasks drawn from common motor performance tools will be used to assess limb coordination: finger-nose, ball strike, ball kick and ball bounce and catch. Finger-nose touch will be performed using the MAND protocol which involves children holding their non preferred arm out in front at shoulder level with the index finger pointed at right angles. The index finger of their preferred hand is used to touch the tip of their nose and the tip of the extended finger moving from supination to pronation. The children will be instructed that it is not a speed test. Ten trials will be performed. Ball strike will be performed using the TGMD-2 Tee ball task which involves a ball being placed on a tee at the height of the child's waist and struck with a bat. The child will be instructed to place their feet shoulder width apart, one on each force plate, facing perpendicular to the intended direction of the t-ball strike and then to hit the ball hard. This task will be repeated 5 times. Ball kick will be performed using the TRMD-2 soccer kick task which involves a ball being positioned on the ground slightly ahead (next to the small force plate) and to the preferred side. Children will be instructed to step forward, from the large force plate onto the small force plate, using their non preferred foot and kick the ball as hard as they can towards a goal. The task will be performed 5 times. The ball bounce and catch task is based on the TGMD-2 ball bounce task and requires the participant to stand on the large force plate and bounce the ball onto the small force plate. Children will be instructed to drop the ball with their preferred hand and catch it with both hands.

Four tasks will be used to assess coordination during active electronic game performance: Move table tennis, Move archery, Kinect table tennis and Kinect soccer penalty kick. For each electronic game task children will be instructed how to perform the task then allowed to practice the task a standard number of times specific to each game. Table tennis will be performed against a computer opponent, with 5 practice points and one game to 11 points repeated on both electronic game consoles. Move archery will be performed against a computer opponent for three trials of 45 seconds. The first set will be used as practice and the final two as assessment trials. The penalty kick trials, performed against a computer opponent, will include one practice trial and three assessment trials.

The primary outcome measures will be the MABC 2 Total Impairment Score, balance as characterised by the length of the path of the centre of mass during the single leg stance trial [28], and upper limb control as characterised by the normalised length of the trajectory of the finger in the finger-nose task [64].

Additionally, movement variability will be assessed using the standard deviation of magnitude and rate of change of kinematics and kinetics across the multiple trials of each task; movement smoothness will be assessed using motion pathway rate of change and jerk [65]; movement efficiency will be assessed using time to stability, out of plane motion and path distance; movement sequencing will be assessed with kinematic chain coupling and time of segmental movement onset; and movement accuracy will be assessed using target error distance. Game performance will also be recorded.

#### Impact of motor coordination on daily living

Parent report of child coordination difficulties interfering with daily life will be assessed with the revised Developmental Coordination Questionnaire (DCDQ-2007) [66], which assesses motor difficulties in individuals from 4 to 15 years of age. It has 15-items divided into three subscales: Control During Movement, Fine Motor/Handwriting and General Coordination, and uses a 5 point Likert scale ranging from 1: 'not at all like your child', to 5: 'extremely like your child'. The DCDQ-2007 is self-administered by parents, comparing their child's motor performance with that of their peers. Using translated versions of the DCDQ-2007 a correlation between test and retest of r = .94 was found (p < 0.001; n = 35) [67]. Factor analysis has verified the three subscales of the DCDQ-2007 explaining 79% of the variance [68], and concurrent validity has been demonstrated with a significant correlation of r = -.55 between the total score on the DCDQ-2007 and the original MABC [68].

#### Physical activity

Time spent in sedentary, light and moderate to vigorous intensity PA, as well as total movement, will be assessed over 7 days using an Actical accelerometer worn on the hip. The Respironics Actical is the most widely used and validated accelerometer in studies of children and adolescents [69]. Seven days of accelerometer measurement are recommended for the purposes of acceptable measurement of moderate to vigorous PA [70]. Total weekly activity as well as weekend activity and after school weekday activity will be assessed. Children will keep a brief diary recording accelerometer wear times and PA to assist with accelerometer data quality control and processing.

#### Attitudes to Physical Activity

Attitudes to PA will be assessed using the revised version [71] of the Physical Activity Enjoyment Scale [72]. Internal consistency, test-retest reliability and construct validity has been demonstrated [71,73,74].

#### Mental Health

Harter's Self-Perception Profile for Children [75] will assess self-perceptions across domains of Scholastic Competence, Social Acceptance, Athletic Competence, Physical Appearance and Behavioural Conduct, and it also includes a subscale designed to evaluate global self-worth that assesses self-esteem independent from the competence domains. It has been validated in samples of children from a wide range of cultural backgrounds, including in Australian children and has high levels of internal consistency ranging from .74 to .92 [75].

Spence Children's Anxiety Scale [76] assesses anxiety symptoms in children and consists of six subscales, namely panic attack and agoraphobia, separation anxiety disorder, social phobia, physical injury fears, obsessive compulsive disorder, and generalized anxiety disorder. This self report questionnaire consists of 45 items, 38 assessing specific anxiety symptoms and the remaining six items serve as positive 'filler' items in order to reduce negative response bias. Children are asked to indicate frequency with which each symptom occurs on a four-point scale ranging from Never (scored 0) to Always (scored 3). A total SCAS score is obtained by summing scores of the 38 anxiety symptom items. The scale has high internal consistency for the total score as well as for each subscale, with satisfactory test-retest reliability [77,78].

The Short Moods and Feelings Questionnaire [79] is a self report screening tool to assess depression in children and adolescents aged 8 to 16, that covers areas such as affective, vegetative and cognitive symptoms of depression. This asks the child to rate depressive symptoms in the past 2 weeks on a Likert scale ranging from 0 to 2, with possible responses of "not true," "sometimes true," and "true". It has 3-week and 3-month test-retest reliabilities of .84 and .80, respectively [80], high internal consistency [80] and validity with depressive diagnoses derived from standardized diagnostic interviews [81].

### Procedure

Following screening, participants will perform baseline assessments at the research centre after informed consent/assent from parent and child. Baseline assessments will include all measures described including laboratory biomechanical measures and questionnaires. Participants will be fitted with the accelerometer for wearing at home for the following week and receive check phone calls reminding the child to wear the accelerometer over the next week and to complete the activity diary. Half the participants will then be randomly allocated to the active electronic game conditions and half to the no electronic game (waitlist/normal care) condition by selection of an opaque sealed envelope. Randomisation will be balanced to equal numbers of boys and girls in both initial condition groups. A research officer (RO) will visit the home after one week and collect the accelerometers and instruct parent and child in the game condition. This will involve either removal of all active electronic games or setting up active electronic game equipment and instructing parent and child in its use. Follow-up phone calls will be made regularly to check participation, whether active game equipment is working correctly and electronic game exposure. Towards the end of the each condition the RO will visit again to set up the accelerometer assessments. After 16 weeks the child and parent will repeat assessments at the research centre. The RO will visit the participants and set up the other game condition. After 16 weeks in the other game condition the same assessments will occur. The family structure including number, age and sex of siblings will be recorded, and the behaviour of siblings during the trial will be assessed at debriefing interview. Accelerometer assessment will be scheduled to avoid school and public holidays where possible. Individualised reports will be provided to participants.

### Trial flow

**Figure 4:**
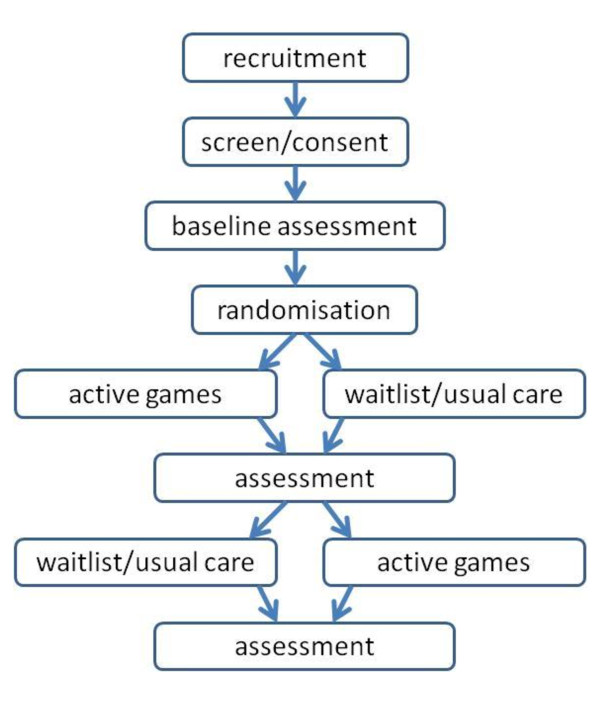
**Trial flow chart showing cross-over design and assessment points**.

Figure 4 provides an overview of the trial flow. Following recruitment, screening and consent and baseline assessments, participants are randomised to an order of electronic game conditions. Participants are setup in their first condition and are assessed at the end of the 16 week condition. Participants are then set up in their second condition for 16 weeks, again with assessment occurring at the end of the condition.

### Analysis

To examine hypothesis 1 (that motor coordination will be improved when children have access to active electronic games) changes from baseline in laboratory biomechanical and MABC-2 outcomes following both conditions will be compared with a repeated measures ANCOVA analysis with sex as a covariate. A critical alpha level of 0.01 will be used to balance type 1 and type 2 errors.

To assess hypotheses 2, 3, 4, 5 (that parent reports of motor coordination in daily living, physical activity and sedentary behaviour, attitudes to physical activity and measures of mental health will improve when children have access to active electronic games) changes from baseline in outcomes following both conditions will be compared with a repeated measures ANCOVA analysis with sex as a covariate. A critical alpha level of 0.01 will be used to balance type 1 and type 2 errors.

## Discussion

Children's increasing use of computers and especially electronic games may reduce their exposure to the large movement activities necessary for normal motor development and engagement in physical activity. Children with Developmental Coordination Disorder may be especially adversely affected. Persuading children to stop playing computer games is unlikely to be successful. However changing the nature of the movements they use when playing games may reduce the negative impact and potentially provide positive gains in motor ability, motor confidence and overall physical activity levels for children with normal and impaired motor development.

### Implications

This project will be the first to assess the longitudinal impact of providing VR electronic games to children with impaired motor development. The knowledge gained from this study will allow us to understand the potential of active VR games to provide children with the motor development and physical activity necessary for a healthy start to life.

## Abbreviations

ANCOVA: analysis of covariance; DCD: developmental coordination disorder; DCDQ: developmental coordination disorder questionnaire; MABC2: movement assessment battery for children - 2; MAND NDI: McCarron assessment of neuromotor disorder neurodevelopmental index; PA: physical activity; RO: research officer; SCAS: Spence children's anxiety scale; TGMD: test of gross motor development; TIS: total impairment scale; VR: virtual reality.

## Competing interests

The authors declare that they have no competing interests.

## Authors' contributions

All authors have contributed substantially to this protocol. LMS conceived the study, contributed to the study design and drafted the manuscript. ACC, LMJ, DRM, AJS, RAA, CMP and JPP contributed to the study design and revised the manuscript. All authors have read and approved the final manuscript.

## Pre-publication history

The pre-publication history for this paper can be accessed here:

http://www.biomedcentral.com/1471-2458/11/654/prepub
